# Transcriptomic Evidence of Adaptive Evolution of the Epiphytic Fern *Asplenium nidus*

**DOI:** 10.1155/2019/1429316

**Published:** 2019-12-01

**Authors:** Jiao Zhang, Li Liu, Jiang-Ping Shu, Dong-Mei Jin, Hui Shen, Hong-Feng Chen, Rui Zhang, Yue-Hong Yan

**Affiliations:** ^1^Shanghai Chenshan Plant Science Research Center, Shanghai Chenshan Botanical Garden, Chinese Academy of Sciences, Shanghai 201602, China; ^2^Shanghai Chenshan Plant Science Research Center, Institute of Plant Physiology and Ecology, Shanghai Institutes for Biological Sciences, Chinese Academy of Sciences, Shanghai 200032, China; ^3^University of Chinese Academy of Sciences, Beijing 100049, China; ^4^Key Laboratory of Plant Resources Conservation and Sustainable Utilization, South China Botanical Garden, Chinese Academy of Sciences, Guangzhou 510650, China

## Abstract

Epiphytic ferns have been found to flourish after angiosperms dominated forest communities, and they play important roles in rainforest canopies. How do epiphytic ferns adapt to tropical rainforest canopy habitats? At present, we know little about the molecular mechanism underlying this adaptation. *Asplenium nidus* is a well-known epiphytic fern that is closely related to the terrestrial species *Asplenium komarovii*. Here, RNA-seq and comparative transcriptomic analyses were performed to explore the underlying basis of the adaptation of *A. nidus* to extreme environments. A total of 44.04 and 44.57 Mb clean reads were obtained from *A. nidus* and *A. komarovii*, respectively, and they were assembled into 89,741 and 77,912 unigenes. Functional annotation showed that 52,305 (58.28% of the total genes for *A. nidus*) and 45,938 (58.96% of the total genes for *A. komarovii*) unigenes were annotated in public databases. Genes involved in stress responses and photosynthesis were found to have undergone positive selection in *A. nidus*. Compared to *A. komarovii*, transcription factors related to stress response, leaf development, and root development were found to be considerably expanded in *A. nidus*, especially in the ANR1 subclade of MADS-box family genes which played roles in lateral root development. This study improves our understanding of the adaptation of *A. nidus* to epiphytic habitats by forming unique strategies.

## 1. Introduction

With 20,000-25,000 species, vascular epiphytes present widely in many families in ferns, gymnosperms, and angiosperms [1, 2]. Epiphytes play important ecological roles in rainforests [3, 4]. Ferns are the second largest group of vascular plants, of which 2,800 species are epiphytic. As a unique group, epiphytic ferns account for one-third of the leptosporangiate ferns, and they can endure severe drought stress, nutrient shortage, and intense sunlight, but not frost [1, 5]. Several epiphytic ferns, including some species in the genera *Vittaria*, *Pyrrosia*, *Polypodium*, and *Platycerium*, have been found to exhibit features typical of crassulacean acid metabolism (CAM) in photosynthesis [6–8]. Epiphytic plants utilize specialized ecological strategies to adapt to variable environments, especially drought stress during dry seasons.

*A. nidus* is a well-known C3 epiphytic fern, and phylogenetic analyses have revealed that it belongs to the *Neottopteris* clade, which is the sister of the *Phyllitis* clade containing the terrestrial species *A. komarovii* (*A. scolopendrium* L. subsp. *Japonicum*) [9–11]. These two species have considerable similarities in morphology, including the presence of lanceolate simple leaves spirally tightly clustered into a bird's nest fern. However, *A. nidus* usually grows to a large size on trees or rocks within the canopy, while *A. komarovii* is not epiphytic and remains small, growing only on the forest floor. The close relatedness but difference in habitat makes these ferns ideal models for comparative genomics and evolution studies of epiphytes.

Comparative genomics (transcriptomics) involves characterizing the differences in gene expression, and it is commonly used in studies of adaptive evolution or evolutionary developmental biology [12–14]. Moreover, transcriptomic data are widely used to study nonmodel organisms [15–17]. In this study, we generated and annotated de novo transcriptome assemblies for both *A. nidus* and *A. komarovii*. Comparative analyses were performed to identify (1) unique genes expressed by *A. nidus*, (2) gene families that have undergone significant expansion, and (3) genes that are under positive selection. We aim to provide new insights on the transcriptomic mechanisms by which epiphytic ferns adapt to canopy habitats in tropical rainforests.

## 2. Materials and Methods

### 2.1. Sample Collection and Transcriptome Sequencing

*A. nidus* and *A. komarovii* were cultivated in a greenhouse of Shanghai Chenshan Botanical Garden (Shanghai, China). Young sporophylls of each species were collected and snap frozen in liquid nitrogen. High-quality total RNA was extracted using TRIzol reagent (Thermo Fisher Scientific, Waltham, MA, USA) following the manufacturer's instructions [18]. After cDNA library preparation, RNA-seq was performed on the Illumina HiSeq 2500 platform at the Beijing Genome Institute (Shenzhen, China). Raw reads of both *A. nidus* and *A. komarovii* were deposited into the NCBI short read archive under accession numbers SAMN11175064 and SAMN11175065.

### 2.2. De Novo Assembly and Functional Annotation

Raw reads were filtered using the Dynamic Trim function in SolexaQA [19] at a quality threshold of 20. Reads shorter than 50 bp were removed. After the filtering, 6.61 and 6.69 G of clean data were obtained from *A. nidus* and *A. komarovii*, respectively. Trinity [20] was used for de novo transcriptome assembly with default settings except that “min_kmer-cov = 2” to reduce the number of error-containing kmers. Thereafter, TGICL 2.1 [21] was utilized to remove the redundant contigs.

The largest contigs were treated as candidate unigenes and used for subsequent analyses. All unigenes were annotated by searching NCBI nonredundant protein database (Nt), the NCBI nonredundant nucleotide database (Nr), the NCBI nonredundant nucleotide database (Nr), the Swiss Institute of Bioinformatics (Swiss-Prot), InterPro, the Gene Ontology database (GO), and the Kyoto Encyclopedia of Genes and Genomes (KEGG). BLAST searches were performed against the Nt, Nr and the Swiss-Prot databases with an *e* value cut-off of 10-5. Thereafter, protein domains were annotated using InterProScan5 [22]. GO annotation was performed by Blast2GO [23]. Transcription factors (TFs) were identified by HMM v3 [24] querying of the PlantTFDB v3.0 database (http://plntfdb.bio.uni-potsdam.de/v3.0/).

BUSCO v3.0.2 (Benchmarking Universal Single-Copy Orthologs) [25] was utilized to assess the completeness of the transcriptome assembly by using a core set of conservative orthologs in eukaryotic species from the OrthoDB database (https://busco.ezlab.org/datasets/).

### 2.3. Identification of Orthologous Genes, Phylogenetic Analysis, and Analysis of Genes under Positive Selection

OrthoFinder v2.3.3 was used to identify orthologs in four fern species (*A. nidus*, *A. komarovii*, *A. formosae*, and *Goniophlebium niponicum*), and transcriptome data were downloaded from the *GigaScience* repository, *Giga*DB [26, 27]. To identify genes under positive selection in *A. nidus*, orthologroups with single-copy genes were retained for further phylogenetic analysis. Amino acid sequences of each orthologroups were aligned using MUSCLE v3.8.31 with default parameters [28]. Maximum likelihood trees were constructed using RAxML v8 with the PROTGAMMAIJTTF model based on the identified orthologous genes [29]. Further, we applied the improved branch-site model in codeml of the PAML v4.8 package on each orthogroups [30]. *A. nidus* was set as the foreground branch, and we calculated the rates of nonsynonymous substitutions (Ka) and synonymous substitutions (Ks). The likelihood ration test and chi-square test were applied to test for significance. Ka < Ks suggested negative (purifying) selection, and Ka > Ks indicated positive selection [31]. In this study, we retained genes with Ks > 0.005; genes with a Ka/Ks ratio greater than 1 were considered putative positively selected genes.

### 2.4. Functional Enrichment

We performed GO enrichment analysis to examine the functional genes involved in the adaptive evolution of *A. nidus*. This analysis was performed in agriGO v2.0 using the singular enrichment analysis tool with hypergeometric's test, as well as by clusterProfiler in R software; Fisher's exact tests (*P* < 0.05) were used to identify genes that were enriched in *A. nidus* compared with *A. komarovii* [32, 33].

### 2.5. Identification and Phylogenetic Analysis of MADS-Box Family Genes

We identified putative MADS-box proteins in *A. nidus* and *A. komarovii* by hidden Markov model (HMM) analysis. Firstly, BLASTP was performed against the protein database of *A. nidus* or *A. komarovii* using *Arabidopsis* MADS-box proteins as queries; the cut-off *e* value for these searches was 1*e* − 05. Secondly, proteins with SRF-TF domains (PF00319) were obtained from the Pfam database, and a HMM analysis was used to identify members of the MADS-box TF family present in *A. nidus* or *A. komarovii*. We verified the identity of the MADS-box candidate genes using SMART (the Simple Modular Architecture Research Tool: http://smart.embl-heidelberg.de/) and the NCBI Conserved Domain Database. Sequences with incomplete MADS-box domains and redundant sequences (with identities higher than 99%) were removed.

To clarify the evolutionary relationships among the MADS-box family genes, 107 *Arabidopsis* MADS-box coding sequences and 49 new candidate MADS-box genes identified in *A. nidus* and *A. komarovii* were aligned using ClustalX v2.1 and a phylogenetic tree was constructed with MrBayes v3.2 using the mixed model (the number of generations as set to 10,000 and the sampling frequency was set to ten). We added generations and maintained the sampling frequency until the standard deviation of split frequencies was below 0.01. The final model contained ten million generations and a tree sampling density of 10,000 generations [34]. The top 25% of samples was discarded as the burn-in. Thereafter, we constructed a phylogenetic tree using all candidate MADS-box protein sequences from *A. nidus*, *A. komarovii*, and *Arabidopsis thaliana* (*Arabidopsis*) containing a conserved MADS domain. Next, phylogeny of type II MADS-box proteins—which contained conserved MADS-, I, and K-domains—was analyzed.

## 3. Results

### 3.1. De Novo Assembly, Completeness Assessment, and Annotation

We sequenced the transcriptomes of *A. nidus* and *A. komarovii* by RNA-seq, and obtained 44.04 and 44.57 Mb clean reads, respectively. Trinity was used for the de novo assembly of 173,229 and 142,138 contigs in *A. nidus* and *A. komarovii* transcriptomes (Table 1). After the redundancies were removed, these transcriptome assemblies yielded 89,741 and 77,912 unigenes in *A. nidus* and *A. komarovii*, with N50 values of 1,314 bp and 1,872 bp, respectively.

We used BUSCO to assess the completeness and quality of the transcriptome assemblies by using a eukaryotic species database containing 429 orthologroups as the reference. These transcriptomes showed high coverage rates of the orthologroups—68.3% in *A. nidus* and 77.7% in *A. komarovii* (Table 1)—indicating that the transcriptomes were relatively complete, and that the data was of high quality and could be used for subsequent analyses.

Functional annotation of the transcriptomes was performed using data from seven public databases. A total of 52,305 (58.28% of the genes in the transcriptome) and 45,938 (58.96%) unigenes were successfully annotated in *A. nidus* and *A. komarovii*, respectively (Table 2). Detailed information on the functional annotation is listed in Table 2.

### 3.2. Comparative Analysis of Unigenes

**Figure 1:**
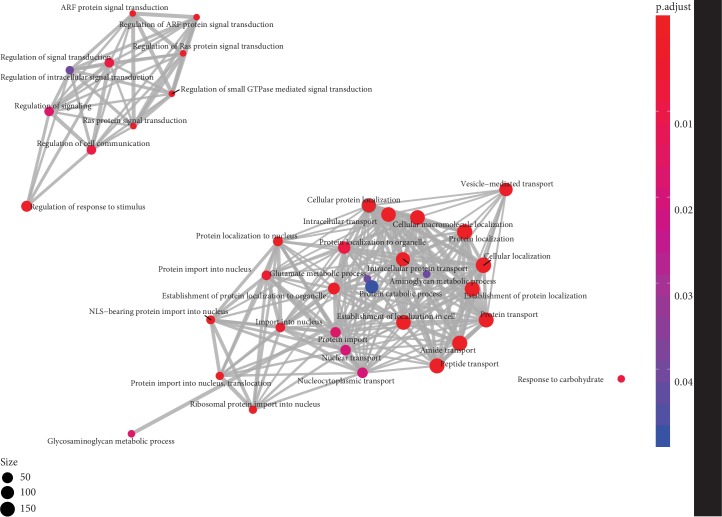
GO enrichment map for *A. nidus*. GO terms, visualized as dots, and shared genes are linked with gray lines.

We compared the orthologous genes present in both *A. nidus* and *A. komarovii*. A total of 20,064 orthologroups were shared by these species, including 25,022 and 27,748 genes in each transcriptome. In addition, we identified 18,160 and 8,970 *A. nidus*- and *A. komarovii*-specific genes. Subsequently, we performed GO enrichment analysis of genes from *A. nidus* (Figure 1, Tables [Supplementary-material supplementary-material-1]). We enriched 38 and 45 GO terms by clusterProfiler and agriGO in *A. nidus* (*P* < 0.05), respectively, in which 24 terms were enriched by both methods. For instance, the regulation of the response to stimulus GO term (GO: 0048583, 51 genes) was enriched only in *A. nidus*. This GO term covered six genes related to abscisic acid synthesis (ABA), and six genes associated with photosynthesis ([Supplementary-material supplementary-material-1]). Taken together, our results revealed that the *A. nidus*-specific genes were mainly involved in the regulation of the response to stimulus and related to photosynthesis, stress tolerance, and ABA signalling.

**Figure 2:**
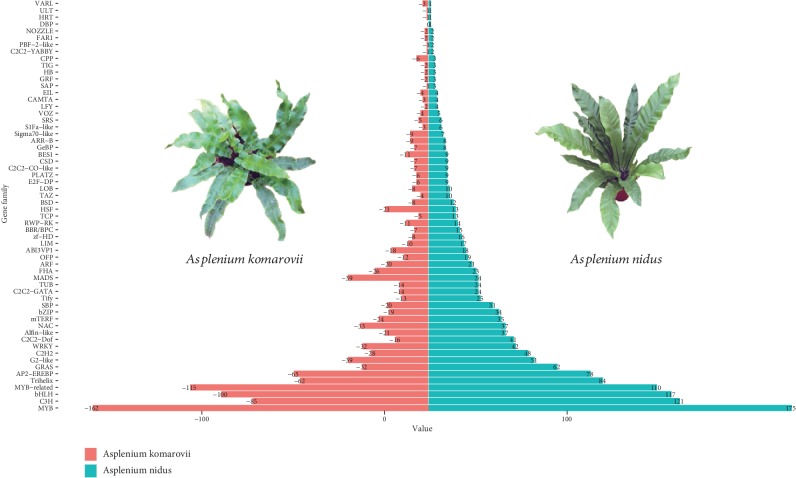
The distribution of TF gene families in *A. nidus* and *A. komarovii*. Blue columns represent TF genes in *A. nidus*, and red columns represent TF genes in *A. komarovii*.

Next, we identified TFs present in *A. nidus* and *A. komarovii* transcriptomes by querying sequences against PlantTFDB. A total of 1,528 and 1,198 putative TF coding unigenes were identified in *A. nidus* and *A. komarovii*, respectively (Figure 2).

Several TFs, such as LIM, TCP, zinc finger-related (including Zf-HD, C2C2-Dof, C2H2, and C3H), and MYB-family TFs, were significantly more abundant in *A. nidus* than in *A. komarovii*. These TFs are mainly involved in leaf and root development, nitrogen assimilation, and plant stress response; therefore, they may be related to the adaptation of *A. nidus* to its epiphytic environments. Moreover, we found that the MADS-box family TFs were less abundant in *A. nidus* than in *A. komarovii*.

### 3.3. Genes under Positive Selection Were Annotated

We used the modified branch-site model in PAML to identify genes under positive selection from the 20,064 orthogroups. As a result, nine genes showed significant evidence of positive selection ([Supplementary-material supplementary-material-1]). These genes were mainly related to environmental adaptability, which included environmental responses and pressure stimulation (i.e., stress-associated protein CL11175 and zinc finger protein CL6726, genes involved in stress tolerance and photosynthesis (i.e., CL12383)).

### 3.4. Identification and Phylogenetic Analysis of MADS-Box Family Genes

**Figure 3:**
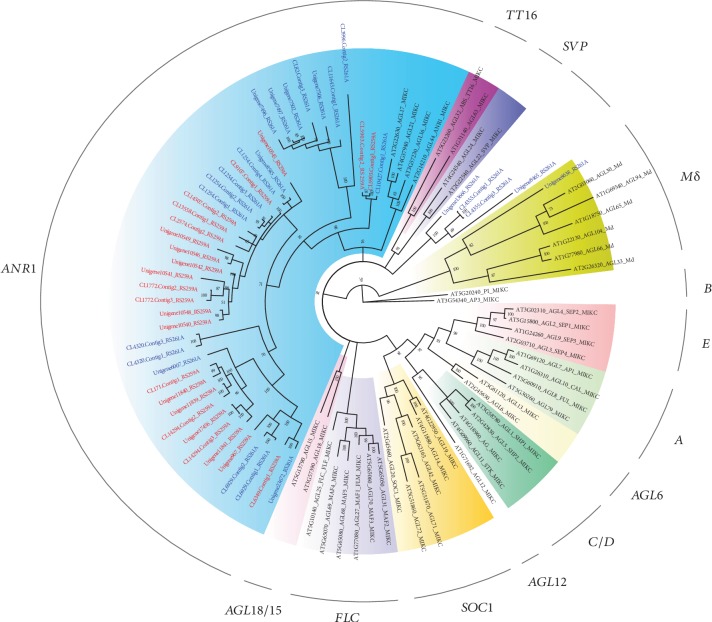
Phylogenetic tree of type II MADS-box proteins in *Arabidopsis*, *A. nidus*, and *A. komarovii*. At, *Arabidopsis*; RS259, *A. nidus*; RS261, *A. komarovii*. In total, 13 clades were formed and marked with different colours.

With extremely specialized roots, *A. nidus* can readily colonize rainforest trees and maintain growth even in very dry conditions. In this way, the roots of *A. nidus* are very different from those of terrestrial ferns such as *A. komarovii*. In *Arabidopsis*, MADS-box genes in ANR1- and AGL12-subclades are involved in root development and differentiation [35]. Given the difference in the copy number of MADS-box family genes in *A. nidus* and *A. komarovii*, we identified and characterized MADS-box family genes in these two species. HMM analysis and manual searching identified 24 and 49 MADS-box family genes in *A. nidus* and *A. komarovii*, respectively. After removing the redundant sequences and genes with incomplete MADS-box domains, a total of 24 and 25 MADS-box family genes were subjected to further phylogenetic analyses. To clarify the evolutionary relationships among the MADS-box family genes, we constructed a phylogenetic tree using the amino acid sequences of MADS-box family genes in *A. nidus*, *A. komarovii*, and *Arabidopsis*. Phylogenetic analysis ([Supplementary-material supplementary-material-1]) revealed that one gene from *A. komarovii* clustered with M*α*-type *Arabidopsis* genes, but we did not find any type I MADS-box family genes in *A. nidus*. With respect to type II MADS-box genes, the MIKC proteins were further divided into 13 well-characterized subclades (Figure 3). One gene was identified as a MIKC∗-type gene in *A. komarovii*. In addition, *A. komarovii* was found to contain four SVP subclade genes; however, these genes were absent in *A. nidus*. Interestingly, 24 genes in the ANR1 subclade were found to have expanded in *A. nidus* compared to 19 genes in *A. komarovii*.

## 4. Discussion

Epiphytes are unique plants that grow on other plants, and they are widely distributed in temperate and tropical rainforests [1, 5]. *A. nidus*, the bird's nest fern is present in tropical rainforests on tree trunks or rocks, whereas *A. komarovii* is a terrestrial species that thrives in temperature regions. *A. nidus* is considered to be more sensitive to climate fluctuations and could endure severe drought stress, nutrient shortage, and intense sunlight [5, 36, 37]. Several epiphytic ferns exhibit features typical of CAM photosynthesis—an adaptative mechanism to dry habitats [6–8]. Previous studies have suggested that large plants, thick fronds, and robust root systems are the major physiological adaptations to drought, although similar analyses of the adaptive features of pure C3 plants have not yet been performed [38–40]. In this study, we identified the factors affecting environmental adaptability in epiphytic *A. nidus* using transcriptomic data. First, we identified genes unique to *A. nidus*, including those related to the regulation of response to stimulus (GO: 0048583, 51 genes). These genes are mainly involved in abiotic stress tolerance, ABA, and photosynthesis; they may contribute to the adaptation of *A. nidus* to drought stress and intense sunlight. Second, we identified genes under positive selection. These genes are usually associated with adaptation [41]. We found nine genes under positive selection in *A. nidus* (Ka/Ks > 1; [Supplementary-material supplementary-material-1]). Among these genes, three were related to environmental adaptability, including stress-associated protein responses, as well as photosynthesis. Previous studies have shown that *OsAKT1* plays essential roles in the ability of K+ channels to uptake in rice; K has crucial roles in various physiological processes, including photosynthesis, assimilated products transport, and tolerance to biotic or abiotic stresses [35, 42]. These genes may also be responsible for the adaptation of *A. nidus* to extreme environments and intense sunlight. Comparisons of the TFs between these two species revealed a significant expansion of the zinc finger-related and MYB gene families in *A. nidus* (Figure 2). The zinc finger-related and MYB gene families were previously reported to be involved in drought tolerance via ABA signalling [43–45]. Given that these TF gene families involved in ABA signalling and plant responses to drought stress, it is likely that *A. nidus* utilizes ABA signalling-mediated pathways to adapt to the epiphytic lifestyle in the tropical rainforest. In addition, genes related to photosynthesis were identified in *A. nidus* by the functional annotation of specific genes and the analysis of genes under positive selection. These genes may contribute to the adaptation of *A. nidus* to intense sunlight [46].

Compared to terrestrial species that take root in soil, epiphytes colonize the crowns of forest trees and face challenges in obtaining water and nutrients [39]. *A. nidus*, a well-known epiphytic fern, has been hypothesized to endure dry conditions by making use of a unique root system and large fronds [38, 47]. It possesses sponge-like roots to absorb and store water, as well as a mass of scaly hairs to protect the apical meristem. In addition, it utilizes its fronds to form basket-shaped rosettes to intercept humus, generate suspended organic soil, and improve nutrient availability.

Significant expansion of gene families is known to be correlated with the adaptive evolution of closely related species [48, 49]. To study the mechanisms that facilitate the absorption of water, we examined genes related to root and leaf development. Comparisons of TFs showed that members of the MADS-box, LIM, and TCP TF families varied considerably between *A. nidus* and *A. komarovii*. Specifically, the ANR1 subclade exhibited a considerable expansion in *A. nidus* (24 members) compared to *A. komarovii* (19 members). However, this conclusion should be cautious because of the transcriptome data used. The higher number of ANR1 genes in *A. nidus* may be related with its higher assembly completeness (52,305 and 45,938 in *A. nidus* and *A. komarovii*, respectively). Nevertheless, BUSCO results showed high coverage rates of the orthologroups in *A. nidus*. At this point, further analyses and complete data are needed to support our speculation. In seed plants, the ANR1 proteins regulate lateral root development [50, 51]. The expanded ANR1-family genes identified here may have acquired novel root development functions via neofunctionalization. The LIM family has been reported to be associated with primary and lateral root development [52, 53]. In addition, class II plant-specific TCP TFs are known to affect local patterns of cell proliferation and to control morphological traits that determine evolutionary success, including leaf formation and shoot branching [54]. These observations suggested that the expansion of gene families related to root or leaf development in *A. nidus* had facilitated its adaptation to drought. More importantly, it is apparent that the epiphytic *A. nidus* has evolved specific strategies with respect to photosynthesis, root development, and frond morphology to withstand extreme canopy environments.

## 5. Conclusions

Due to its epiphytic growth habitat, *A. nidus* has been forced to withstand extreme environments. In this study, we constructed de novo transcriptome assemblies for *A. nidus* and its close relative *A. komarovii*. Comparative transcriptomic analysis showed that genes unique to *A. nidus* were mainly involved in stress tolerance and photosynthesis, implying that these genes may contribute to its adaptation to drought stress and intense sunlight. It is notable that the expansion of TF gene families and *A. nidus*-specific genes was related to ABA signalling pathway and stress responses, which potentially reflect the adaptation of *A. nidus* to drought. The expansion of TF gene families related to root or leaf development may have also facilitated this adaptation. Overall, our data suggest that *A. nidus* has evolved specific adaptations related to photosynthesis, root development, and large frond morphology to withstand the extreme epiphytic environment. There are also important limitations to our study. Firstly, the transcriptome represented only a portion of all coding genes; therefore, it provided limited sequence information due to the fact that genes are dynamically expressed. Secondly, tissues of *A. nidus* and other species should be sampled to verify these findings. Lastly, more detailed functional experiments are needed to provide deeper insights into the molecular mechanisms of epiphytic adaptations to harsh environments.

## Figures and Tables

**Table 1:** Summary statistics of the completeness assessments of *A. nidus* and *A. komarovii* transcriptome assemblies.

ID	Species	Total number of clean reads (Mb)	Total number of unigenes	Mean length (bp)	N50 value (bp)	BUSCOs notation assessment results
RS259A	*Asplenium nidus*	44.04	89,741	770	1,314	*C*: 68.3% [*S*: 32.4% *D*: 35.9%], *F*: 13.5%, *M*: 18.2%, *n*: 429
RS261A	*Asplenium komarovii*	44.57	77,912	1,049	1,872	*C*: 77.7% [*S*: 42.7%, *D*: 35.0%], *F*: 7.5%, *M*: 14.8%, *n*: 429

C: complete BUSCOs; S: complete and single-copy BUSCOs; D: complete and duplicated BUSCOs; F: fragmented BUSCOs; M: missing BUSCOs; n: total number of BUSCO groups searched.

**Table 2:** Functional annotation results of *A. nidus* and *A. komarovii* transcriptomes.

Database	*Asplenium nidus*		*Asplenium komarovii*	
Total	89,741	100%	77,912	100%
Overall	52,305	58.28%	45,938	58.96%
Nt	25,472	28.38%	25,050	32.15%
Nr	47,800	53.26%	41,178	52.85%
COG	17,509	19.51%	16,985	21.80%
Swiss-Prot	33,121	36.91%	28,148	36.13%
InterPro	34,663	38.63%	31,923	40.97%
GO	17,871	19.91%	14,801	19.00%
KEGG	36,164	40.30%	31,253	40.11%

Note: the two columns represent the number of unigenes and the percentage of annotated unigenes.

## Data Availability

The data used to support the findings of this study have been deposited in the NCBI short reads archive repository under accession numbers SAMN11175064 (*A. nidus*) and SAMN11175065 (*A. komarovii*).

## Supplementary Materials

Supplementary 1 Figure S1: the phylogenetic tree of MADS-box genes in *Arabidopsis thaliana*, *Asplenium* nidus, and *Asplenium komarovii* constructed with MrBayes.

Supplementary 2 Table S1: the enrichment of specific genes in *A. nidus*.

Supplementary 3 Table S2: the functional annotation of specific genes in *A. nidus*.

Supplementary 4 Table S3: GO annotation of positively selected genes in *A. nidus*.
