# Supplementary figures and images for: Transcriptomic Evidence of Adaptive Evolution of the Epiphytic Fern Asplenium nidus
Source: Int J Genomics. 2019 Dec 1;2019:1429316. doi: 10.1155/2019/1429316 (PMC6913284; doi:10.1155/2019/1429316)

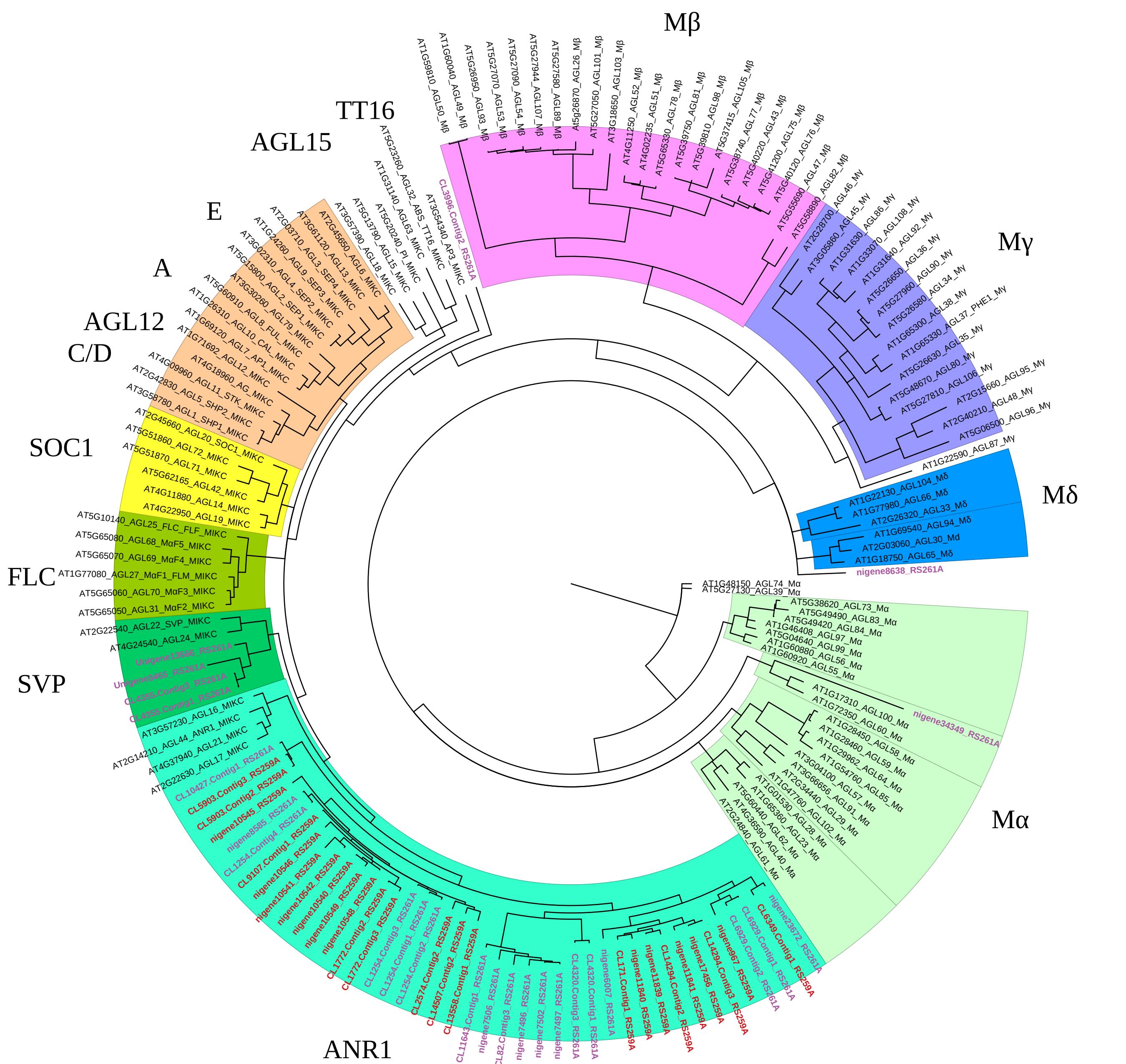

Supplement: Supplementary 1 — Figure S1: the phylogenetic tree of MADS-box genes in Arabidopsis thaliana, Asplenium nidus, and Asplenium komarovii constructed with MrBayes. [file 1429316.f1.pdf]
